# Monitoring of circulating monocyte HLA-DR expression in a large cohort of intensive care patients: relation with secondary infections

**DOI:** 10.1186/s13613-022-01010-y

**Published:** 2022-05-08

**Authors:** C. de Roquetaillade, C. Dupuis, V. Faivre, A. C. Lukaszewicz, C. Brumpt, D. Payen

**Affiliations:** 1 Anesthesiology and Critical Care Unit, Hopital Lariboisière, Paris, France; 2 Medical Intensive Care Unit, Clermont-Ferrand University Hospital, 58 rue Montalembert, 63003 Clermont-Ferrand, France; 3 Anesthesiology Laboratory, Hospital Lariboisière, Paris, France; 4 Anesthesia & Critical Care, Hospital Neurologique, Hospices Civils de Lyon, EA 7426: Pathophysiology of Injury-Induced Immunosuppression (PI3), Université de Lyon 1, Lyon, France; 5 Service d’Hématologie Biologique, Pôle B2P, Hôpital Lariboisière, APHP, Paris, France; 6 Université Paris 7 UFR de Médecine, 110 A venue de Verdun, 75010 Paris, France; 7 INSERM U942, Département MASCOT, 43 Boulevard de la Chapelle, 75010 Paris, France

**Keywords:** Septic shock, ICU immunodepression, Innate immunity, HLA-DR, Intensive care patients, Immune monitoring

## Abstract

**Introduction:**

The reports of an early and profound acquired immunodepression syndrome (AIDs) in ICU patients had gained sufficient credence to modify the paradigm of acute inflammation. However, despite several articles published on AIDs and its assessment by monocytic HLA-DR monitoring, several missing informations remained: 1—Which patients’ are more prone to benefit from mHLA-DR measurement, 2—Is the *nadir* or the duration of the low mHLA-DR expression the main parameter to consider? 3—What are the compared performances of leukocytes’ count analyses (lymphocyte, monocyte).

**Material and method:**

We conducted an observational study in a surgical ICU of a French tertiary hospital. A first mHLA-DR measurement (fixed flow cytometry protocol) was performed within the first 3 days following admission and a 2nd, between day 5 and 10. The other collected parameters were: SAPS II and SOFA scores, sex, age, comorbidities, mortality and ICU-acquired infections (IAI). The associations between mHLA-DR and outcomes were tested by adjusted Fine and Gray subdistribution competing risk models.

**Results:**

1053 patients were included in the study, of whom 592 had a 2nd mHLA-DR measurement. In this cohort, 223 patients (37.7%) complicated by IAI. The initial decrement in mHLA-DR was not associated with the later occurrence of IAI, (*p* = 0.721), however, the persistence of a low mHLA-DR (< 8000 AB/C), measured between day 5 and day 7, was associated with the later occurrence of IAI (*p* = 0.01). Similarly, a negative slope between the first and the second value was significantly associated with subsequent IAI (*p* = 0.009). The best performance of selected markers was obtained with the combination of the second mHLA-DR measurement with SAPSII on admission. Persisting lymphopenia and monocytopenia were not associated with later occurrence of IAI.

**Conclusion:**

Downregulation of mHLA-DR following admission is observed in a vast number of patients whatever the initial motif for admission. IAI mostly occurs among patients with a high severity score on admission suggesting that immune monitoring should be reserved to the most severe patients. The initial downregulation did not preclude the later development of IAI. A decreasing or a persisting low mHLA-DR expression below 8000AB/C within the first 7 days of ICU admission was independently and reliably associated with subsequent IAI among ICU patients with performances superior to leukocyte subsets count alone.

**Supplementary Information:**

The online version contains supplementary material available at 10.1186/s13613-022-01010-y.

## Introduction

The frequent reports of an early and profound acquired immunodepression syndrome (AIDs) in ICU patients had gained sufficient credence to modify the paradigm of acute inflammation [1]. Even the definition remains debated, the quite constant element is a severe alteration of antigen-presenting cells (APC) to present the antigen to lymphocytes [1]. The association of altered lymphocyte absolute number with a reduction of their functional panel is frequently associated [2–4]. If the mechanisms are not fully elucidated at a molecular level, its detection can be accurately made by different methods such as ex vivo stimulation and cytokine response [5], ex vivo assessment of PBMCs’ phagocytic capabilities [6] and longitudinal monitoring of circulating monocytes HLA-DR (mHLA-DR) [5, 7, 8]. Despite numerous articles published on AIDs with a mHLA-DR reduction including in severe COVID-19 [9, 10], several missing informations can be obtained only on a large cohort of ICU patients admitted for different diagnoses, using a homogenous flow cytometry protocol to measure mHLA-DR. The present retrospective study aimed to answer the following question: 1—Which patients’ typologies are more prone to benefit from mHLA-DR measurement, 2—Is the nadir or the duration of the low mHLA-DR expression the main parameter to consider? 3—what is the real link between low mHLA-DR and the occurrence of secondary infections? Solving these issues might help clinicians to decide what patient would have to be monitored [11] and potentially to be stimulated using available drugs (interferon γ anti-PD-1, GMCSF, IL-7) [12, 13] for untreatable opportunistic infections [11]. The clarification of these questions is essential to validate the role of immune dysfunction and to design clinical trials to test the benefit of additional immunomodulatory therapies.

## Material and methods

This study was approved by Cochin Hospital Ethics Committee (# CCPPRB 2061, Assistance Publique Hôpitaux de Paris). The mHLA-DR blood tests did not require the patient's informed consent since it was a retrospective study with routine measurements in our institution performed on the remaining routine blood samples, with a guarantee to use the data after their anonymization, according to the Ethical French law.

### Study design and population

The cohort of patients was collected on the patients’ database admitted in our center between 2013 to 2015 having routine measurements of HLA-DR, to evaluate the meaning of mHLA-DR monitoring. The only criteria used to select the cohort was to have had at least one measurement of mHLA-DR performed within the first 3 post ICU admission days. Patients hospitalized for less than 3 days, moribund, or treated with a chronic treatment by immunosuppressive drugs were excluded. Based on the motif for admission, 4 main clusters of life-threatening conditions were observed: (1) sepsis, defined by the criteria of the American College of Chest Physicians/Society of Critical Care Medicine [14]; (2) neurologic disorders, related to acute brain injury such as hemorrhagic or ischemic stroke; isolated severe brain trauma; post-neurosurgery; (3) major surgery (abdominal, orthopedic, ENT); (4) miscellaneous etiologies including primary respiratory failure, hemorrhagic shock from gastrointestinal bleeding or obstetric emergencies. IAI was diagnosed using the classic definition [15, 16]: a new-onset infection starting at least 48 h after ICU admission, which motivated a new antimicrobial therapy. The likelihood of infection motivating the clinical decision to administer antibiotics was classified as none, possible, probable, and definite [15]. Details of the classification method are provided in the e-Method section of Additional file 1. At the time of secondary infection diagnosis, the in-charge physician was not aware of the mHLA-DR value. Two senior intensivists (first and last author) blindly reviewed all patients’ medical charts and adjudicated all secondary infections. In case of discordance, a third expert settled the final diagnosis (CD).

### Circulating monocyte HLA-DR measurements (mHLA-DR)

The quantification of the expression of HLA-DR on monocytes was assessed using the number of antibodies per cell (AB/C) by flow cytometry (FACS Canto II instrument, FACS Diva software, Becton Dickinson, San Jose, CA USA) as previously described [7] (see detailed protocol in the e-method section of Additional file 1). In our center, the median and IQ range of mHLA-DR expression in healthy people for the measurements at the same period (*n* = 13) was a median log mHLA-DR value of 40,134 (IQR: 36,315–44,353). The first blood sample and measurement of mHLA-DR were performed within the first 3 days after admission. The 2nd measurement of mHLA-DR was obtained on fixed days (Monday or Thursday) until the patient’s discharge or death. Since we used a survival model, only measurements sampled before the event were considered for analyses, and data were blinded at the time of the event (death or IAI). We used the threshold of AB/C < 8000 to define “low mHLA DR” corresponding to the acquired immune suppression as previously reported (NCT02361528) and because it corresponded to the median value observed in previously published datasets [7, 12]. Only the first nosocomial infection was considered for analysis.

### Statistical analysis

The data were described as number and percentage for categorical variables and median (interquartile range (IQR)) for continuous variables. Comparisons relied on the Fisher exact test or *χ*^2^ test for categorical data and the Kruskal–Wallis or Wilcoxon test for continuous data. Because of non-linearity, all the mHLA-DR values were log-transformed. Age and SOFA scores were categorized based on the median value. A *p*-value of less than 0.05 was considered statistically significant.

Standard survival analyses are affected by the time of onset of the event of interest. Patients who have not experienced the event at the end of follow-up were censored. To determine the risk of an event occurring at a certain time-point, a fundamental assumption is that such censoring is not associated with an altered chance of the event occurring at any given moment. In this study, the event of interest is the occurrence of nosocomial infections and followed up until day 28 or until leaving alive from ICU. Indeed, death and leaving alive from ICU are competing events since, by definition, extubation precludes the observation of a ventilator-associated infection [17]. For that purpose, the association between mHLA-DR measurements and outcomes was assessed using adjusted Fine and Gray subdistribution competing risk models [18]. We first took into account the competing ICU discharge for the subdistribution hazard of mHLA-DR measurements on death at day 28. The subdistribution hazard of mHLA-DR measurements on the occurrence of IAI at day 28 was made considering the competing ICU death and ICU discharge. For each model, risk factors for the different outcomes were first researched by univariate analyses. The covariates tested into the models were the following: age, motif of admission, SOFA on day 1, comorbid conditions, and immune suppression. Although parenteral nutrition and the use of a central venous catheter are usual risk factors for NI, they were excluded from the predictors of NI. Almost every patient was managed with a central venous catheter, and enteral nutrition only was given to our patients. Then, the variables yielding *p*-values < 0.2 in univariate analysis were entered into a multivariate model using a backward selection, with *p* < 0.05 considered significant. The mHLA-DR measurement was forced into all the models. Results were expressed as subdistribution hazard ratios (sHR) with their 95% confidence intervals (95% CIs).

To analyze other immune factors possibly associated with IAI, we performed the same analyses using lymphocyte count (with lymphopenia defined as lymphocyte count 1000/mm^3^) and monocyte count (with monocytopenia defined as monocyte count below 500/mm^3^).

We assessed the robustness of our findings using multiple sensitivity analyses. We performed internal validation using a bootstrapping procedure, which was done by taking a large number of samples of the original one. This technique provides nearly unbiased estimates of the confidence intervals (CI) of the odds ratio (OR) of the independent covariates. Second, we performed logistic regression sensitivity analysis. Third, we used a multivariate cause-specific survival model. Fourth, we analyzed previous immune suppression as a comorbid condition and included age into the model. Fifth, we provide sensitivity analyses focusing on documented secondary infections and more specifically for secondary infections occurring at least 48 h after the second mHLA-DR measurement. Similar testing was applied for the VAP. All analyses were performed using SAS software, version 9.4 (SAS Institute Inc., Cary, North Carolina).

## Results

### Description of the cohort

Among the screened 1766 patients admitted in our ICU during the study period, 1053 patients benefited from a measurement of mHLA-DR within first days of admission. Among them, 592 patients benefitted from a second measurement and were included for secondary infection analyses (for flowchart, see Additional file 1: Fig. S1). Motifs for admission were: isolated brain injury (*n* = 384, 36.5%); sepsis (*n* = 255, 24.2%); major surgery (*n* = 80, 7.6%); miscellaneous diagnoses (*n* = 334, 31.7%) (Table 1). Overall, ICU-mortality was 14.3% (*n* = 151). One episode IAI was diagnosed in 223 patients (37.7%) (Table 1) with a median delay from admission of 7 days (IQR [5; 11]). Secondary infections were mostly VAP (*n* = 126, 56.5%), abdomen nosocomial infections (peritonitis, biliary tract) (*n* = 40, 17.9%) and bacteremia/catheter-related infections (*n* = 35, 13.3%). The rate of these IAI was higher after major surgery (*n* = 28, 48.8%) and brain injury (*n* = 109, 43.95%) than after sepsis (*n* = 54, 31.8%, *p* < 0.01) (Additional file 1: Table S1).

**Table 1:** Characteristics of the global cohort and patients having 2 measurements of mHLA-DR

Parameters, *n* (%) or median [IQR]	All patients (*n* = 1053)	Patients with two mHLA-DR measurement (*n* = 592)
Age	59.3 [44.8; 71.8]	59.8 [46.5; 71.4]
Sex (female)	444 (42.2)	236 (39.9)
Comorbid condition		
Hypertension	399 (37.9)	197 (39.6)
Cardiac insufficiency	233 (22.1)	100 (20.1)
Immunosuppression	229 (21.7)	94 (18.9)
Diabetes	155 (14.7)	81 (16.3)
Respiratory failure	98 (9.3)	46 (9.3)
Chronic kidney disease	91 (8.6)	39 (7.8)
Cirrhosis	47 (4.5)	17 (3.4)
Diagnostic on admission		
Sepsis	255 (24.2)	170 (28.7)
Septic shock (classic definition)	77 (7.3)	72 (42.4)
Origin of the infection		
Cutaneous	83 (32.5)	61 (35.9)
Respiratory	65 (25.5)	46 (27.1)
Abdominal	61 (23.9)	36 (21.2)
Neurologic	15 (5.9	11 (6.5)
Urinary	18 (7.1)	9 (5.3)
Others	13 (5.1)	7 (4.1)
Neurologic admission	384 (36.5)	248 (41.9)
Subarachnoid hemorrhage	103 (26.8)	75 (30.2)
Brain Traumatism	68 (17.7)	49 (19.8)
Intra cranial hemorrhage	68 (17.7)	42 (16.9)
Neurological surgery	63 (16.4)	32 (12.9)
Ischemic stroke	50 (13)	27 (10.9)
Subdural hematoma	18 (4.7)	10 (4)
Others	17 (4.4)	13 (5.2)
Post-surgical care	80 (7.6)	41 (6.9)
Abdominal	59 (73.8)	33 (80.5)
ORL	8 (10)	5 (12.2)
Orthopedics	8 (10)	2 (4.9)
Others	5 (6.3)	1 (2.4)
Miscellaneous	334 (31.7)	133 (22.5)
Hemorrhagic shock	67 (20.1)	30 (22.6)
Respiratory failure from a medical origin	79 (23.7)	27 (20.3)
Polytrauma	37 (11.1)	25 (18.8)
Medical abdominal disease	31 (9.3)	13 (9.8)
Obstetrical	24 (7.2)	6 (4.5)
Cardiac arrest	13 (3.9)	5 (3.8)
Others	83 (24.9)	27 (20.3)
Severity on admission		
Day 1 SAPS II (miss = 6)	37 [25; 50]	39 [28; 51]
Day 1 SOFA (neuro excluded) (miss = 6)	4 [2; 7]	5 [2; 8]
HLA-DR Cell count measurements		
Delay from admission to mHLA-DR measurement, days (1st/2nd)	2 [1; 3]	2 [1; 3]/5 [4; 7]
Log mHLA-DR (1st/2nd)	9.2 [8.7; 9.7]	9.1 [8.7; 9.6]/9.2 [8.7; 9.6]
Low mHLA-DR, < 000 AB/C (1st/2nd)	403 (38.3)	253 (42.7)/221 (37.3)
Leucocytes, 10^9^/L (1st/2nd)	10.8 [8.5; 14.4]	11.1 [8.9; 15.1]/11 [8.6; 4.5]
Neutrophils, 10^9^/L (1st/2nd)	8.5 [6.4; 11.8]	9 [6.6; 12.5]/8.6 [6.2; 11.7]
Lymphocytes, 10^9^/L (1st/2nd)	1.3 [0.9; 1.7]	1.2 [0.8; 1.7]/1.3 [1; 1.8]
Monocytes, 10^9^/L (1st/2nd)	0.7 [0.5; 1]	0.8 [0.5; 1.1]/0.8 [0.6; 1.1]
Outcomes		
Delay before 1st ICU-acquired infection (days)	7 [5; 10.5]	7 [5; 11]
Number of ICU-acquired infections	245 (23.3)	223 (37.7)
Source of ICU-acquired infection		
Respiratory	138 (13.1)	126 (21.3)
Abdominal	44 (4.2)	40 (6.8)
Bacteremia, catheter-related	40 (3.8)	35 (5.9)
Others	23 (2.2)	22 (3.7)
ICU length of stay	7 [4; 14]	13 [8; 22]
Delay before death	6 [3; 14]	13 [8; 25]
ICU death	151 (14.3)	79 (13.3)
Early death, (< day 7, *n* = 1040)	82 (7.9)	–
Late ICU death (among patients alive at day 7, *n* = 499)		60 (12)

IQR: interquartile; ICU: Intensive Care Unit; SAPS: Simplified Acute Physiology Score; SOFA: Sequential Organ Failure Assessment; NLCR: neutrophil-to-lymphocyte count ratio; mHLA-DR: monocytic human leukocyte antigen-antigen D related; AB/C: antibody per cell. For diagnostic at admission, data are expressed as percentage within the subgroups

### mHLA-DR measurement at presentation

**Fig. 1:**
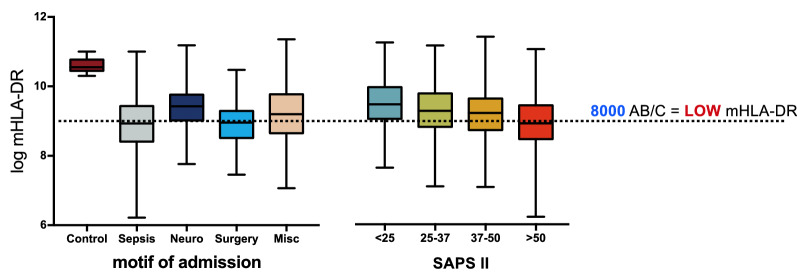
Initial mHLA-DR measurement according to the motif of admission and initial severity, comparison with controls. Low mHLA-DR expression is defined by a level < 8000AB/C

The 1st mHLA-DR expression median value was 9.2 log (IQR 8.7–9.7), with an average delay for measurement of 2 [1; 3] days. The number of patients having a low mHLA-DR < 8000 AB/C at baseline (defined as the threshold for low mHLA-DR) was 38.3% (*n* = 403). mHLA-DR expression values for all clusters were lower than those obtained from healthy volunteers and was observed among all prespecified subgroups of admission (Fig. 1). mHLA-DR downregulation was associated with initial severity assessed by the SAPS II, *R*^2^ = − 0.28 (IC95% [− 0.34 to − 0.23], *p* < 0.01).

### Relation between mHLA-DR kinetic and later occurrence of IAI

**Fig. 2:**
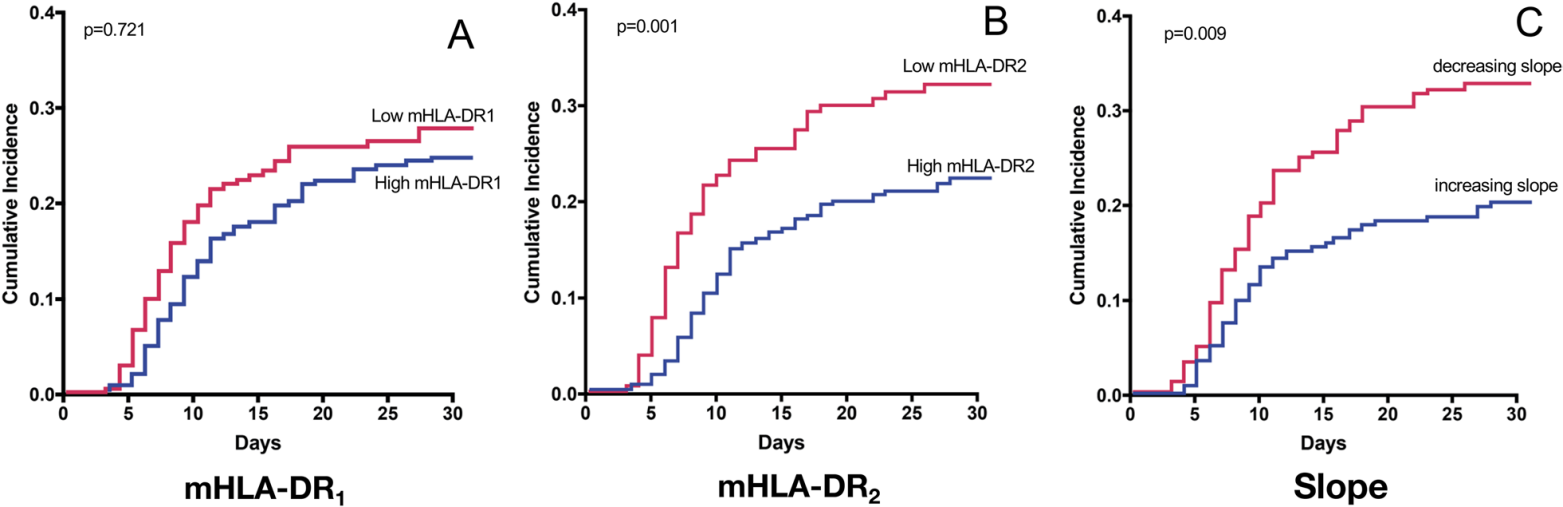
Cumulative incidence of the occurrence of nosocomial infection depending on different levels of early mHLA-DR expression (**A**), second mHLA-DR expression (**B**), and depending on the trend of mHLA-DR (**C**) in patients with two measurements (*n* = 592). Low mHLA-DR expression is defined by a level < 8000. *p*-value estimated by a multivariate subdistribution survival model

**Fig. 3:**
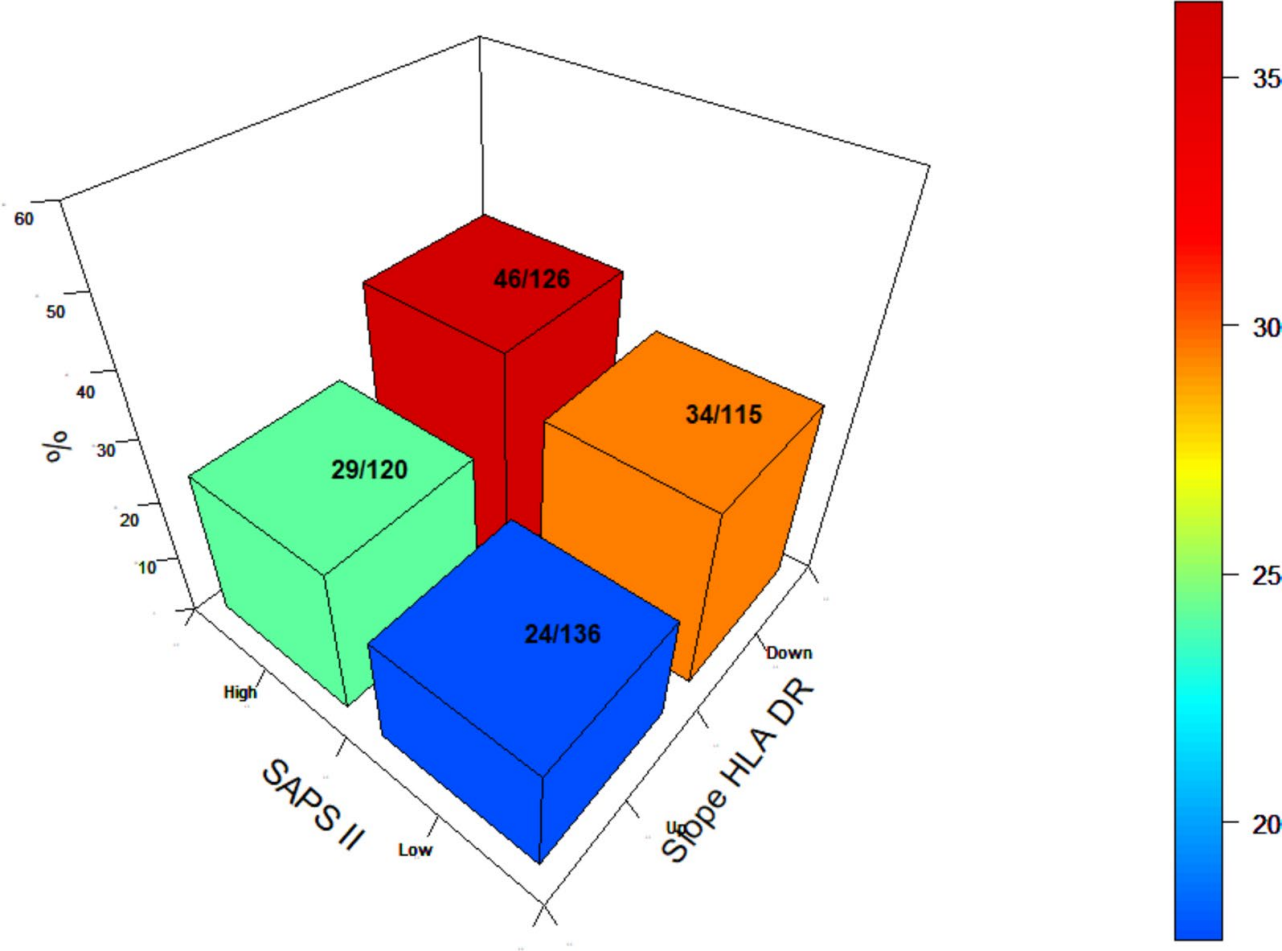
Tridimensional representation of the relation between SAPS II—the slope of mHLA-DR and the occurrence of secondary infection at day 28. Figure represents patients dichotomized based on median SAPS II and mHLA-DR > or ≤ to 8000 AB/C. Whatever initial gravity, the existence of a decreasing slope between the first and second measurement of mHLA-DR is a strong risk factor for the later occurrence of ICU-acquired infection. SAPS II: Simplified Acute Physiology Score 2; mHLA-DR: monocytic human leukocyte antigen-antigen D related

A second mHLA-DR measurement was performed in 592 of the 1053 patients (Table 1). Those patients were mainly admitted for brain injury (*n* = 248, 41.9%) and sepsis (*n* = 170, 28.7%). In this cohort, the initial decrement in mHLA-DR was not associated with the later occurrence of IAI, after adjustment for confounding factors (*p* = 0.721) (Fig. 2). However, the persistence of a low mHLA-DR (< 8000 AB/C), measured between day 5 and day 7, was associated with the later occurrence of IAI (*p* = 0.01). Similarly, a negative slope between the first and the second value was significantly associated with subsequent IAI (*p* = 0.009). When SAPS II was combined with the negative mHLA-DR slope, IAI incidence was higher than with each component alone (Fig. 3).

In the cause-specific multivariate model (Table 2), the first measurement of mHLA-DR < 8000 AB/C was not associated with subsequent secondary infection (cs-HR 1.02 [0.7; 1.48], *p* = 0.93), however, a decreasing slope between first and second mHLA-DR measurement was associated with subsequent occurrence of secondary infection (cs-HR 1.53 [1.06; 2.19], *p* = 0.02). The combination of a first low mHLA-DR and decreasing slope was strongly associated with later occurrence of secondary infection (cs-HR 1.73 [1.07; 2.82], *p* = 0.03). Lymphopenia within the first days of admission was associated with the later occurrence of IAI, this was not the case for its persistence nor the decrease in lymphocyte count during ICU stay. The decreased monocyte count was negatively associated with delayed occurrence of IAI (cs-HR = 0.66 [0.47; 0.92], *p* = 0.02). Overall, the best performance of selected markers was obtained with the combination of the second mHLA-DR measurement with SAPSII on admission (AUC 0.62 IC95% [0.56–0.67]) (Additional file 1: Table S2).

**Table 2:** Multivariate cause-specific survival model: sensitivity analysis of predictors of secondary infection occurrence

Variable	Cs-HR	IC95%	*p*
Log mHLA-DR1	0.90	[0.67; 1.19]	0.46
Low mHLA-DR 1	1.06	[0.72; 1.57]	0.77
Log mHLA-DR2	0.63	[0.48; 0.81]	0.00
Low mHLA-DR2	1.75	[1.21; 2.53]	0.00
Slope (%)	0.98	[0.97; 0.99]	0.00
Slope (↘)	1.61	[1.13; 2.3]	0.01
Low mHLA-D1 and down slope	1.95	[1.17; 3.26]	0.01
Lymphopenia 1	1.62	[1.13; 2.32]	0.01
Lymphopenia 2	0.87	[0.58; 1.3]	0.51
Lymphopenia down	0.98	[0.69; 1.41]	0.93
Monocytopenia 1	0.94	[0.65; 1.34]	0.71
Monocytopenia 2	0.59	[0.41; 0.85]	0.00
Monocytopenia down	0.66	[0.47; 0.92]	0.02

The cause-specific model is a common alternative to survival analysis for handling competing risks. This model allows a quantification of the cause-specific relative hazard, which is the association between the exposure and the outcome when the individuals with the competing event are censored

The following covariates were used: comorbidities (without immunosuppression), immunosuppression, SOFA score and motif of admission

The competing event was “discharged alive before 28 days” and the outcome was nosocomial infection

Using bootstrap sensitivity analysis, the association between the negative slope of mHLA-DR and later occurrence of IAI remained significant (HR = 1.53, IC95% [0.99; 2.38], Additional file 1: Table S3a). A logistic regression sensitivity analysis to predict IAI occurrence retrieved a strong association between negative slope and IAI (OR 1.74, CI95% [1.14; 2.68]) (Additional file 1: Table S3b). Subgroup analyses retrieved that the association between decreasing mHLA-DR and subsequent IAI was mostly driven by the septic patients subgroup (Additional file 1: Table S4).

Since the second measurement of mHLA-DR may be influenced by the acquired infection itself particularly if not yet detected, we performed a sensitivity analysis focusing on IAI occurring at least 48 h after last mHLA-DR measurement (Additional file 1: Table S5). Among the 458 patients identified for this analysis, a decrement in mHLA-DR (decreasing slope) remained associated with later development of IAI (HR = 2.16, IC95% [1.39–3.36], *p* < 0.01). Analysis focusing on documented NI (*n* = 251/276) retrieved similar results (HR = 1.86, IC95% [1.27; 2.74], *p* < 0.01) for mHLA-DR_2_ < 8 000AB/C and HR = 1.57, IC95% [1.07; 2.29], *p* = 0.02 for decreasing slope). However, association between mHLA-DR value or kinetic and subsequent VAP was not significant (HR = 1.19, IC95% [0.66; 2.16], *p* = 0.56 for mHLA-DR_2_ < 8 000AB/C and HR = 1.66, IC95% [0.96; 2.86], *p* = 0.07 for decreasing slope).

## Discussion

### Key results

In this observational monocentric large cohort of ICU patients, the monitoring of mHLA-DR during the first-week post-admission showed a strong association between persisting low expression of mHLA-DR and the further development. An early mHLA-DR downregulation was observed in a large proportion of patients whatever the initial motif for admission suggesting a common pathway of resilience to aggression [19, 20]. The clear correlation between the severity at admission and the depth of mHLA-DR downregulation indicates that such monitoring might be indicated for the most severe patients. Our study supports the interest to repeat the monitoring of mHLA-DR expression during the first post-admission days to identify the patients at risk for IAI with a threshold of 8000 AB/C to define an ICU-acquired immune suppression when consensus protocol for measurement is applied.

### Interpretations

The steps for generalization of immune biomarkers to identify ICU patients at risk of complications as IAI require large cohorts and validation by randomized clinical trials. We and others have previously reported similar results in reasonable cohorts of ICU patients, mostly septic [7, 8, 10, 21, 22]. The present study confirms these previous results and investigated different ICU contexts, including septic, surgical and neurologic patients [7]. Altogether these findings confirm the adapted early downregulation of mHLA-DR (as a resilience mechanism) [19, 20] to maintain the tissue fitness and limit the consequences of acute inflammation. Conversely to most previous reports, our analyses was made after adjustments for confounding factors as severity and occurrence of IAI as potential downregulating mHLA-DR. Moreover, it took into account the comorbid conditions, particularly the previous immune suppression. The present study highlights the risk of a persisting mHLA-DR downregulation as a marker of immune suppression and its association with the increased vulnerability to IAI [1]. The other markers proposed to diagnose AID had a limited reproducibility and performance when compared to HLA-DR [22]. In addition, these markers necessitate specific human skills to be performed and are not feasible on a day-to-day basis to help for clinical decision. In the present study, the leucocyte absolute number especially the lymphocytes absolute number were not as informative as mHLA-DR was. A recent article reports the longitudinal testing of injury-induced immune profile changes (30 immune biomarkers) in a large cohort of ICU patients [23]. Except for T cell and CD4 T cell absolute number, none of the 30 markers were significantly different between sepsis, trauma or surgical groups, suggesting a “universal phenomenon”, which does not depend on the type of injury as reported for HLA-DR [7]. Among the leading markers, CD74 mRNA and mHLA-DR seemed to have the best performance to assess an IAI in relation with secondary infection. Interestingly, mHLA-DR + S1009A were the best predicators for secondary infection. Such “multimodal immune monitoring approach” deserves further prospective study to tailor the immunomodulating therapies.

The perspective of such immune monitoring is to objectively help the clinician to characterize the innate immunity and the immune synapse with adaptive immunity, to characterize the AID and to propose to stimulate innate immunity, as reported previously [12]. The persistence of AID associated with IAI despite adequate antimicrobial therapy may then justify using immunostimulating drugs as it was shown in recent articles [12, 24–27]. Enrolling the adequate patients in the futures clinical trials will then benefit from such immune monitoring, markedly the mHLA-DR that fits well to the requirements of ICU clinical context. Based on our observation, we can suggest that immune monitoring should be reserved to the most severe patients.

### Limitations

The current study has, however, some limitations. First, it is a monocentric evaluation, which hampers the generalization of the results to all centers. Second, no other immune parameters, such as cytokines plasma levels or NK cells, or Treg lymphocytes have been measured. Even with this limitation, this was coherent with our choice for routine immune monitoring in the “real life” in our center. Third, based on our results, no prospective cohort of patients has been used to prospectively test the validated parameters. We try to limit this aspect by the use of the bootstrap statistical method, which confirmed the primary analysis. Some limitations might also be seen as advantages. The care protocols were more homogenous within one center and the measurement of mHLA-DR was stable for the protocol and rigorously measured following the European task force [28]. The technical variation in the measurements may then result only from the manual steps of the protocol, which will be solved by the development of automated devices. The collected cohort covered clinical contexts with systemic inflammation in the “real life” corresponding to our recruitment with limited exclusion. To our knowledge, the size of our cohort is the largest reported for now that confirmed previous results obtained in smaller cohorts [7, 10]. The occurrence of an early AID was observed in the vast majority of the ICU patients [7, 8, 29]. The deliberate choice to not consider the patients dying before day 4 post-admission was coherent with the goal of the study. The blood sampling for mHLA-DR as early as the first 2 days was useful for testing the kinetic of evolution as this potential marker to predict IAI, which was shown to be relatively stable within the first 4 post-admission days [10].

When considering only VAP, mHLA-DR value and kinetic was not associated with subsequent VAP (*p* = 0.07). Several assumptions may be made to explain such results. Diagnosis of VAP is very difficult, and may be missed by both clinical examination and radiological exam. Several studies have acknowledged important inter-observatory variability in the diagnosis of ICU-acquired infection. Despite the use of external review and CDC criteria, as in our case, postmortem studies comparing VAP diagnosis with clinical criteria showed 69% sensitivity and 75% specificity, in comparison to autopsy findings [30]. On the contrary, diagnosis of bacteremia and/or post-surgery infection is less debatable. VAP also carries a specific pathophysiology which not only relies on immunological factors, but also on local factors (micro-inhalation, reintubation, exposure to ventilator), which unfortunately, could not be taken into account in our model.

## Conclusion

A rapid decrease in mHLA-DR within the first days following admission is observed in a vast number of patients whatever the initial motif for admission. Initial downregulation of mHLA-DR correlates with the severity on admission suggesting that immune monitoring should be applied to the most severe patients. The initial decrement in mHLA-DR does not preclude the later development of IAI, whereas a decreasing or a persisting low mHLA-DR expression below 8000 AB/C within the first 7 days of ICU admission was independently and reliably associated with subsequent IAI among ICU patients The performance of these parameters is superior to leukocyte subsets count alone.

## Supplementary Information

**Additional file 1.** Supplementary material comprises e-method, flow chart and sensitivity analyses

## Data Availability

The data and materials will be made available upon motivated request addressed to the corresponding author.
