# Supplementary material for: Monitoring of circulating monocyte HLA-DR expression in a large cohort of intensive care patients: relation with secondary infections
Source: Ann Intensive Care. 2022 May 8;12:39. doi: 10.1186/s13613-022-01010-y (PMC9079217; doi:10.1186/s13613-022-01010-y)
Supplement: Supplementary file 1 — Additional file 1. Supplementary material comprises e-method, flow chart and sensitivity analyses [file 13613_2022_1010_MOESM1_ESM.docx]

**mHLA-DR Additional file**

e-Method

**Flow cytometry protocol**

Supplementary data: The sample was processed within the 2 hours post sampling. The staining was performed using isotype control (Simultest control #1/#2a), anti HLA-DR-PE (clone L243, BD Biosciences) and anti-CD14-FiTC (clone M$P9, Beckman Coulter Immunotech,Marseille, France). The Mean fluorescence intensity (MFI) was converted into antibodies per cell (AB/C). The sample was processed within the 2 hours post sampling. The staining was performed using isotype control (Simultest control #1/#2a), anti HLA-DR-PE (clone L243, BD Biosciences) and anti-CD14-FiTC (clone M$P9, Beckman Coulter Immunotech, Marseille, France). The Mean fluorescence intensity (MFI) was converted into antibodies per cell (AB/C) and PE signal was calibrated every month with Quantibrite kit (BD Biosciences).

**Definition of comorbidities:** Immune suppression was defined as a medical history of immune deficiency, human immune deficiency virus (HIV) or acquired immune deficiency syndrome (AIDS), solid organ transplant, systemic inflammatory disease or autoimmune disease or by the use of corticosteroids and any neoplastic condition past or present. Cardiovascular insufficiency was defined as having a medical history of congestive heart failure, chronic cardiovascular disease, arrythmia, coronary artery disease. Renal insufficiency was defined as a history of chronic renal insufficiency or chronic intermitted hemodialysis or continuous ambulatory peritoneal dialysis. Respiratory insufficiency was defined as chronic obstructive pulmonary disease, asthma or respiratory insufficiency in the medical history. Cirrhosis was defined by the presence of a chronic hepatic failure reported by the patient or in medical charts.

**Definition and criteria for nosocomial infection, adapted from 2005 International Sepsis Forum Consensus Conference on Definitions of Infection in the Intensive Care Unit**

*Pneumonia*

Definite: The patient must have a new or progressive radiographic infiltrate, along with a high clinical suspicion of pneumonia (or a CPIS of ≥6, using a Gram stain of a lower respiratory tract sample) plus a definite cause established by the recovery of a probable etiologic agent from a) an uncontaminated specimen (blood, pleural fluid, transtracheal aspirate, or transthoracic aspirate); b) the recovery from respiratory secretions of a likely pathogen that does not colonize the upper airways (e.g., Mycobacterium tuberculosis, Legionella species, influenza virus, or Pneumocystis jiroveci (carinii); c) recovery of a likely/possible respiratory pathogen in high concentrations using quantitative cultures of a lower respiratory tract sample (endotracheal aspirate, BAL, or protected specimen brush); or d) positive serology.

Probable: The patient must have a new or progressive radiographic infiltrate along with a high clinical suspicion of pneumonia (or a CPIS of ≥6, using a Gram stain of a lower respiratory tract sample) plus detection (by staining or culture) of a likely pulmonary pathogen in respiratory secretions (expectorated sputum, endotracheal or bronchoscopic aspirate, or quantitatively cultured bronchoscopic BAL fluid or brush catheter specimen), but in concentrations below the diagnostic threshold, or the presence of a negative lower respiratory tract culture if collected within 72 hrs after starting a new antibiotic regimen.

**Possible pneumonia defined as abnormal chest radiograph of uncertain cause, in a patient with a low or moderate clinical suspicion of pneumonia were not considered for analyses**

**Bloodstream infection** Patient with a recognized pathogen cultured from blood cultures.

Bloodstream infections included catheter-related sepsis with bacteriologic confirmation, defined as at least one peripheral positive blood culture and one of the following:

● A positive semiquantitative (≥15 colony-forming units [cfu]/catheter segment) or quantitative (≥10^3^ cfu/catheter segment) catheter tip culture (i.e., catheter colonization), whereby the same microorganism (species and antibiogram) is isolated from the catheter segment and peripheral blood

● A positive hub or exit site culture growing the same microorganism as peripheral blood or

● Positive paired central and peripheral blood cultures growing the same organism, where the central blood culture is positive ≥2 hrs earlier than the peripheral blood culture or has five times the growth of the peripheral blood culture

**Abdominal infection** is a microbial infection of the peritoneal space following perforation, abscess formation, ischemic necrosis, or penetrating injury of the intra-abdominal contents Microbiologically confirmed: Isolation of one or more microbial pathogens found in the peritoneum or the blood ≥24 hrs after a gastrointestinal perforation of the stomach, esophagus or duodenum, or any perforation of the small bowel distal to the ligament of Treitz. Spillage of luminal contents during an operative procedure is not sufficient evidence of perforation that allows for definitive diagnosis of peritonitis. Furthermore, a penetrating abdominal wound or documented perforation that is surgically repaired within 12 hrs of its occurrence is not sufficient evidence to support diagnosis of secondary bacterial peritonitis.

Probable: Compatible clinical illness associated with documented evidence of perforation (free air in the abdomen on radiographic studies or surgical confirmation of peritoneal inflammation following luminal perforation in the absence of microbiologically confirmed peritonitis). A Gram stain in the absence of a positive culture from the peritoneum would be considered probable secondary bacterial peritonitis.

Possible: Upper gastrointestinal perforation or penetrating abdominal trauma that is surgically repaired without further evidence of microbiologic confirmation or clinical signs or symptoms supportive of a diagnosis of bacterial or fungal peritonitis. A finding of an inflammatory peritoneal fluid in the presence of a documented but localized intra-abdominal abscess in the absence of culture confirmation would also be considered possible secondary bacterial peritonitis.

**
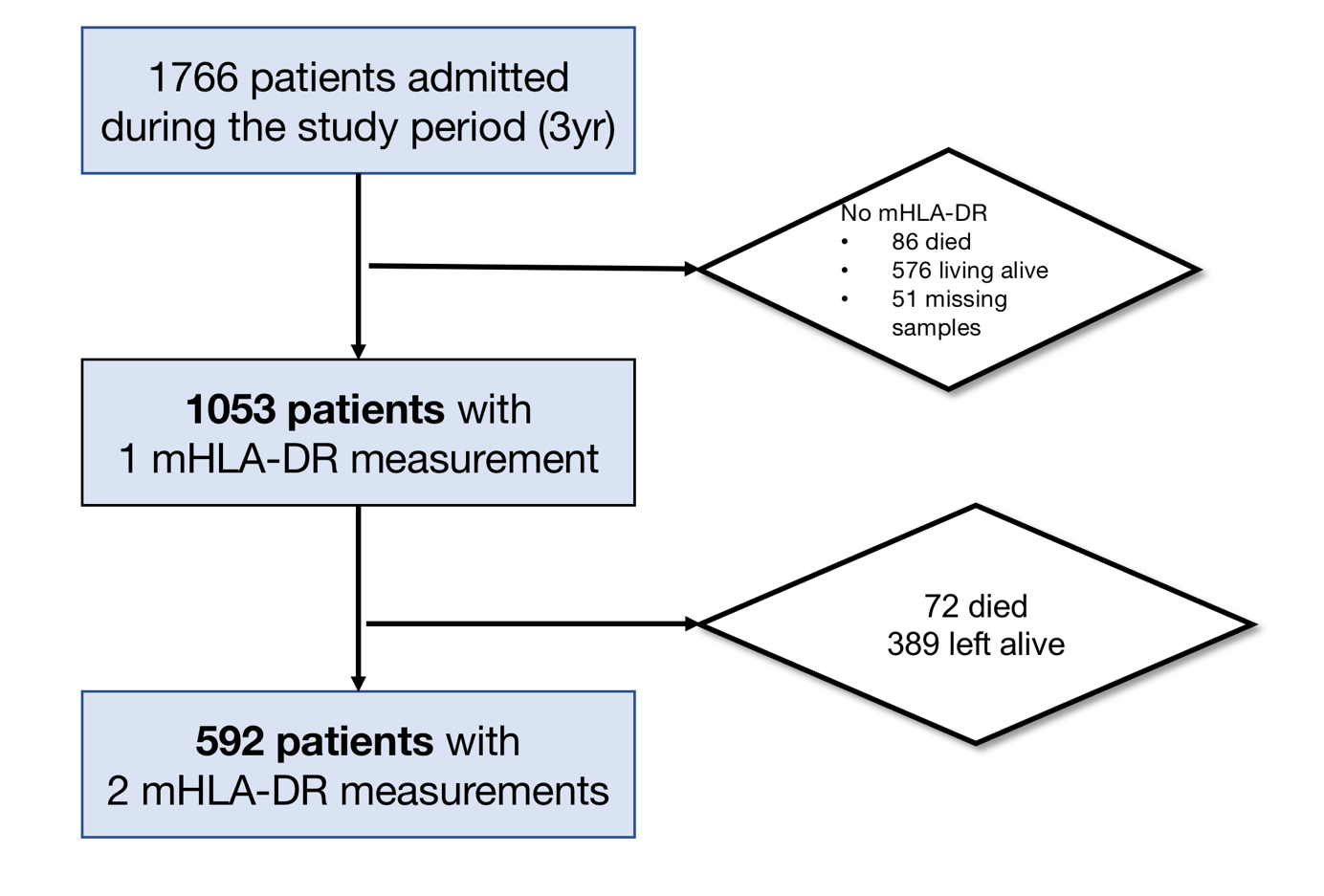
**

Figure S1: flow-chart

mHLA-DR: Monocyte Human Leukocyte Antigen – antigen D Related

**Table S1:** main characteristics of subgroups of patients depending on their motif of admission in the whole cohort (n=1053)

| **Patients characteristics** | **All** | **Sepsis** | **Neurologic** | **Post-surgery** | **Miscellaneous** | **P value** | **N vs M** | **P vs N** | **S vs P** | **P vs M** | **S vs M** | **S vs N** |
| --- | --- | --- | --- | --- | --- | --- | --- | --- | --- | --- | --- | --- |
| **(N(%) or median[IQR])** |  |  |  |  |  |  |  |  |  |  |  |  |
| **Number of patients (N)** | 1053 | 255 | 384 | 80 | 334 |  |  |  |  |  |  |  |
| Age | 59.3 [44.8 ; 71.8] | 66.29 [49.93 ; 79.29] | 54.82 [43.72 ; 64.93] | 62.5 [55.54 ; 69.74] | 60.02 [40.52 ; 74.54] | <0.01 | <0.01 | <0.01 | 0.30 | 0.15 | <0.01 | <0.01 |
| sex (female) | 444 (42.2) | 109 (42.75) | 159 (41.41) | 36 (45) | 140 (41.92) | 0.05 | 0.89 | 0.55 | 0.72 | 0.62 | 0.84 | 0.74 |
| **Comorbid condition** |  |  |  |  |  |  |  |  |  |  |  |  |
| Hypertension | 399 (37.9) | 106 (41.57) | 140 (36.46) | 33 (41.25) | 120 (35.93) | 0.4180 | <.01 | <.01 | <.01 | <.01 | 0.79 | <.01 |
| Cardiac insufficiency | 233 (22.1) | 70 (27.45) | 62 (16.15) | 22 (27.5) | 79 (23.65) | 0.0010 | 0.04 | 0.09 | 0.76 | 0.78 | 0.37 | <.01 |
| Immunosuppression | 229 (21.7) | 65 (25.49) | 31 (8.07) | 51 (63.75) | 82 (24.55) | <.0001 | 0.01 | 0.02 | 0.99 | 0.47 | 0.29 | <.01 |
| Diabetes | 155 (14.7) | 57 (22.35) | 35 (9.11) | 17 (21.25) | 46 (13.77) | <.0001 | 0.65 | 0.52 | 0.26 | 0.62 | 0.10 | 0.18 |
| Respiratory failure | 98 (9.3) | 32 (12.55) | 23 (5.99) | 9 (11.25) | 34 (10.18) | 0.3331 | <.01 | 0.52 | 0.05 | 0.05 | 0.96 | <.01 |
| Chronic kidney disease | 91 (8.6) | 30 (11.76) | 20 (5.21) | 7 (8.75) | 34 (10.18) | 0.0013 | <.01 | <.01 | <.01 | <.01 | 0.14 | <.01 |
| Cirrhosis | 47 (4.5) | 10 (3.92) | 7 (1.82) | 2 (2.5) | 28 (8.38) | 0.0007 | 0.88 | 0.42 | 0.96 | 0.38 | 0.16 | 0.19 |
| SAPSII | 37 [25 ; 50] | 38 [29 ; 52] | 37.5 [27 ; 50] | 29 [21 ; 40] | 38.5 [24 ; 52] | <0.01 | 0.93 | <0.01 | <0.01 | <0.01 | 0.14 | 0.14 |
| SOFA on admission | 4 [2 ; 7] | 5 [2 ; 9] | 4 [2 ; 6] | 3 [1 ; 5] | 4 [2 ; 8] | <0.01 | 0.03 | <0.01 | <0.01 | <0.01 | 0.21 | <0.01 |
| **Cell count and mHLA-DR on admission** |  |  |  |  |  |  |  |  |  |  |  |  |
| Leucocytes (10^9) | 10.8 [8.5 ; 14.4] | 11.9 [8.5 ; 16.8] | 10.8 [8.9 ; 13.65] | 10.45 [7.5 ; 13.65] | 10.5 [8 ; 14.4] | 0.016 | 0.48 | 0.13 | 0.01 | 0.34 | 0.01 | 0.02 |
| Neutrophils (10^9) | 8.5 [6.4 ; 11.8] | 9.88 [6.56 ; 14.2] | 8.3 [6.51 ; 10.77] | 7.58 [5.59 ; 11.52] | 8.47 [6.03 ; 11.53] | <0.01 | 0.94 | 0.32 | <0.01 | 0.41 | <0.01 | <0.01 |
| Lymphocytes (10^9) | 1.3 [0.9 ; 1.7] | 1.14 [0.69 ; 1.6] | 1.37 [1 ; 1.89] | 0.95 [0.63 ; 1.43] | 1.3 [0.9 ; 1.72] | <0.01 | <0.01 | <0.01 | 0.12 | <0.01 | <0.01 | <0.01 |
| Monocytes (10^9) | 0.7 [0.5 ; 1] | 0.7 [0.43 ; 1.05] | 0.81 [0.6 ; 1.1] | 0.57 [0.41 ; 0.94] | 0.72 [0.5 ; 1] | <0.01 | <0.01 | <0.01 | 0.22 | 0.05 | 0.47 | <0.01 |
| NLCR | 7.1 [4.4 ; 11.1] | 8.84 [5.6 ; 14.01] | 6.41 [4.12 ; 9.14] | 8.98 [5.45 ; 12.65] | 6.72 [4.18 ; 11.1] | <0.01 | 0.07 | <0.01 | 0.39 | 0.02 | <0.01 | <0.01 |
| mHLA-DR (log) | 9.2 [8.7 ; 9.7] | 8.93 [8.41 ; 9.43] | 9.43 [9.02 ; 9.76] | 8.96 [8.52 ; 9.29] | 9.2 [8.65 ; 9.77] | <0.01 | <0.01 | <0.01 | 0.92 | <0.01 | <0.01 | <0.01 |
| Low mHLA-DR | 403 (38.3) | 138 (54.12) | 91 (23.7) | 42 (52.5) | 132 (39.52) | <0.01 | <0.01 | <0.01 | 0.80 | 0.03 | <0.01 | <0.01 |
| **Outcome** |  |  |  |  |  |  |  |  |  |  |  |  |
| ICU-acquired infection (first episode) | 245 (23.3) | 57 (22.35) | 117 (30.47) | 22 (27.5) | 49 (14.67) | <0.01 | <0.01 | 0.60 | 0.34 | <0.01 | 0.02 | 0.02 |
| Origin of the nosocomial infection * |  |  |  |  |  |  |  |  |  |  |  |  |
| *Respiratory* | 138 (56.3) | 27 (10.59) | 82 (21.35) | 10 (12.5) | 33 (9.88) | <0.01 | 0.81 | 0.57 | 0.71 | 0.76 | 0.65 | 0.75 |
| *Abdominal* | 44 (18) | 16 (6.27) | 5 (1.3) | 10 (12.5) | 18 (5.39) | <0.01 | 0.13 | 0.37 | 0.12 | 0.28 | 0.18 | 0.20 |
| *Bacteremia* | 40 (16.3) | 15 (5.88) | 16 (4.17) | 4 (5) | 12 (3.59) | 0.42 | 0.77 | 0.61 | 0.70 | 0.25 | 0.39 | 0.78 |
| ICU Length of stay | 7 [4 ; 14] | 8 [5 ; 16] | 10 [5 ; 18] | 6 [3 ; 10.5] | 5 [3 ; 9] | <0.01 | <0.01 | <0.01 | <0.01 | 0.05 | <0.01 | 0.04 |
| ICU Death | 151 (14.3) | 45 (17.65) | 58 (15.1) | 6 (7.5) | 42 (12.57) | <0.01 | 0.33 | 0.07 | 0.03 | 0.20 | 0.09 | 0.39 |

IQR: interquartile; ICU: Intensive care Unit- SAPS: Simplified Acute Physiology Score- SOFA: Sequential Organ Failure assessment; NLCR : Neutrophil-to-lymphocyte count ratio; HLA-DR: Human Leukocyte Antigen – antigen D Related –N: Neurologic – S: Sepsis – M: Miscellaneous – P: Post surgery. * Detail of the total ICU-acquired infections

**Table S2.** Performance analyses of mHLA-DR, lymphocyte count, monocyte count for prediction of outcome, comparison with severity scores alone and in combination.

|  |  |  | AUC | threshold | specificity | sensitivity | accuracy | tn | tp | fn | fp | npv | ppv | 1-specificity | 1-sensitivity | 1-npv | 1-ppv |
| --- | --- | --- | --- | --- | --- | --- | --- | --- | --- | --- | --- | --- | --- | --- | --- | --- | --- |
| ICU-acquired infection | 2 HLA | Log HLA DR t1 | 0.51 [ 0.46 - 0.57 ] | 9.25 | 0.45 | 0.63 | 0.5 | 165 | 84 | 49 | 199 | 0.77 | 0.3 | 0.55 | 0.37 | 0.23 | 0.7 |
|  | 2 HLA | Log HLA DR t2 | 0.6 [ 0.55 - 0.65 ] | 9.63 | 0.32 | 0.86 | 0.46 | 115 | 115 | 18 | 249 | 0.86 | 0.32 | 0.68 | 0.14 | 0.14 | 0.68 |
|  | 2 HLA | Down slope HLA DR | 0.58 [ 0.53 - 0.63 ] | 0.5 | 0.56 | 0.6 | 0.57 | 203 | 80 | 53 | 161 | 0.79 | 0.33 | 0.44 | 0.4 | 0.21 | 0.67 |
|  | 2 HLA | Slope HLA DR | 0.61 [ 0.56 - 0.67 ] | 1.17 | 0.53 | 0.67 | 0.57 | 192 | 89 | 44 | 172 | 0.81 | 0.34 | 0.47 | 0.33 | 0.19 | 0.66 |
|  | 2 HLA | SAPSII | 0.57 [ 0.52 - 0.63 ] | 30.5 | 0.34 | 0.8 | 0.47 | 125 | 107 | 26 | 239 | 0.83 | 0.31 | 0.66 | 0.2 | 0.17 | 0.69 |
|  | 2 HLA | SAPSII/HLA DR t2 | 0.62 [ 0.56 - 0.67 ] | 0.27 | 0.61 | 0.61 | 0.61 | 222 | 81 | 52 | 142 | 0.81 | 0.36 | 0.39 | 0.39 | 0.19 | 0.64 |
|  | 2 HLA | Lymphocytes t1 | 0.56 [ 0.5 - 0.62 ] | 0.82 | 0.77 | 0.37 | 0.66 | 280 | 49 | 84 | 84 | 0.77 | 0.37 | 0.23 | 0.63 | 0.23 | 0.63 |
|  | 2 HLA | Lymphocytes t2 | 0.52 [ 0.46 - 0.57 ] | 1.4 | 0.46 | 0.61 | 0.5 | 167 | 81 | 52 | 197 | 0.76 | 0.29 | 0.54 | 0.39 | 0.24 | 0.71 |
|  | 2 HLA | Lymphocytes t1 | 0.56 [ 0.5 - 0.62 ] | 1 | 0.65 | 0.46 | 0.6 | 238 | 61 | 72 | 126 | 0.77 | 0.33 | 0.35 | 0.54 | 0.23 | 0.67 |
|  | 2 HLA | Lymphocytes t2 | 0.52 [ 0.46 - 0.57 ] | 1 | 0.71 | 0.29 | 0.6 | 258 | 38 | 95 | 106 | 0.73 | 0.26 | 0.29 | 0.71 | 0.27 | 0.74 |
|  | 2 HLA | Monocytes t1 | 0.52 [ 0.46 - 0.58 ] | 0.94 | 0.7 | 0.39 | 0.62 | 254 | 52 | 81 | 110 | 0.76 | 0.32 | 0.3 | 0.61 | 0.24 | 0.68 |
|  | 2 HLA | Monocytes t2 | 0.59 [ 0.53 - 0.65 ] | 0.8 | 0.59 | 0.59 | 0.59 | 213 | 78 | 55 | 151 | 0.79 | 0.34 | 0.41 | 0.41 | 0.21 | 0.66 |
|  | 2 HLA | Monocytes t1 | 0.52 [ 0.46 - 0.58 ] | 0.5 | 0.22 | 0.74 | 0.36 | 79 | 99 | 34 | 285 | 0.7 | 0.26 | 0.78 | 0.26 | 0.3 | 0.74 |
|  | 2 HLA | Monocytes t2 | 0.59 [ 0.53 - 0.65 ] | 0.5 | 0.2 | 0.83 | 0.37 | 71 | 111 | 22 | 293 | 0.76 | 0.27 | 0.8 | 0.17 | 0.24 | 0.73 |

**Table S3a:** Bootstrap sensitivity analysis of predictors of secondary infection among patients with two mHLA-DR measurements.

| **Subgroup** | **Variable** | **HR** | **HR CI 95%** |
| --- | --- | --- | --- |
|  |  |  |  |
| **Patients with 2 measurements** | Log mHLA-DR1 | 1 | [0.72; 1.42] |
|  | Low mHLA-DR1 | 1.02 | [0.63;1.71] |
|  | Log mHLA-DR2 | 0.82 | [0.56; 1.17] |
|  | Low mHLA-DR2 | 1.32 | [0.83;2.14] |
|  | Slope (%) | 0.99 | [0.97; 1.00] |
|  | Slope (↘) | 1.53 | [0.99; 2.38] |
|  | Low mHLA-DR1 & Slope (↘) | 1.73 | [0.85;3.36] |

§All the final models are adjusted on comorbidities (without immunosuppression), immunosuppression, SOFA score and motif of admission.

HR: Hazard Ratio; CI: Confidence interval; ICU: Intensive care Unit; SAPS: Simplified Acute Physiology Score; SOFA: Sequential Organ Failure assessment; HLA-DR: Human Leukocyte Antigen – antigen D Related.

**Table S3b:** Logistic regression sensitivity analysis of predictors of secondary infection occurrence

|  | **Subgroup** | **Variable** | **OR** | **OR CI 95%** |
| --- | --- | --- | --- | --- |
|  |  |  |  |  |
| **Nosocomial infection** | **Patients with 2 measurements** | Log mHLA-DR1 | 1 | [0.73; 1.39] |
|  |  | Low mHLA-DR1 | 0.92 | [0.59;1.50] |
|  |  | Log mHLA-DR2 | 0.66 | [0.48; 0.9] |
|  |  | Low mHLA-DR2 | 1.52 | [0.98;2.36] |
|  |  | Slope (%) | 0.97 | [0.96; 0.99] |
|  |  | Slope (↘) | 1.74 | [1.14; 2.68] |
|  |  | Low mHLA-DR1 & Slope (↘) | 1.88 | [1.02;3.47] |

§All the final models are adjusted on comorbidities (without immunosuppression), immunosuppression, SOFA score and motif of admission.

**Table S4: Subgroup analyses: predictors of the occurrence of death, multivariate sub distribution survival analyses.**

| Table | Variable | Sub HR | Sub HR CI | pvalue |
| --- | --- | --- | --- | --- |
| Neurology | Log mHLA-DR1 | 0.62 | [0.33 ; 1.16] | 0.14 |
| (2 mHLA-DR) | Low mHLA-DR 1 | 1.29 | [0.57 ; 2.96] | 0.54 |
|  | Log mHLA-DR2 | 0.41 | [0.22 ; 0.75] | <0.01 |
|  | Low mHLA-DR2 | 2.30 | [1.02 ; 5.19] | 0.04 |
|  | Slope (%) | 0.99 | [0.96 ; 1.02] | 0.33 |
|  | Slope (↘) | 1.19 | [0.54 ; 2.6] | 0.67 |
|  | Low mHLA-D1 and Down slope | 1.54 | [0.51 ; 4.66] | 0.44 |
|  | Lymphopenia 1 | 1.96 | [0.96 ; 3.97] | 0.06 |
|  | Lymphopenia 2 | 1.33 | [0.63 ; 2.83] | 0.45 |
|  | Lymphopenia down | 1.28 | [0.61 ; 2.68] | 0.52 |
|  | Monopenia 1 | 0.82 | [0.37 ; 1.84] | 0.64 |
|  | Monopenia 2 | 1.09 | [0.48 ; 2.47] | 0.83 |
|  | Monocypenia down | 0.77 | [0.37 ; 1.62] | 0.49 |
| Sepsis | Log mHLA-DR1 | 0.93 | [0.46 ; 1.85] | 0.83 |
| (2 mHLA-DR) | Low mHLA-DR 1 | 0.58 | [0.24 ; 1.42] | 0.23 |
|  | Log mHLA-DR2 | 0.69 | [0.46 ; 1.05] | 0.08 |
|  | Low mHLA-DR2 | 2.67 | [1.08 ; 6.57] | 0.03 |
|  | Slope (%) | 0.95 | [0.91 ; 0.98] | 0.01 |
|  | Slope (↘) | 4.36 | [1.85 ; 10.3] | <0.01 |
|  | Low mHLA-D1 and Down slope | 2.48 | [1.02 ; 6.01] | 0.04 |
|  | Lymphopenia 1 | 1.59 | [0.73 ; 3.46] | 0.24 |
|  | Lymphopenia 2 | 1.69 | [0.76 ; 3.75] | 0.20 |
|  | Lymphopenia down | 1.12 | [0.49 ; 2.55] | 0.79 |
|  | Monopenia 1 | 1.20 | [0.53 ; 2.73] | 0.66 |
|  | Monopenia 2 | 1.01 | [0.46 ; 2.2] | 0.99 |
|  | Monocypenia down | 2.49 | [1.03 ; 6.03] | 0.04 |

All the final models are adjusted on comorbidities (without immunosuppression), immunosuppression, SOFA score and motif of admission.

HR: Hazard Ratio; CI: Confidence interval; ICU: Intensive care Unit; SAPS: Simplified Acute Physiology Score; SOFA: Sequential Organ Failure assessment; HLA-DR: Human Leukocyte Antigen – antigen D Related.

**Table S5. Sensitivity analysis focusing on patients with mHLA-DR measurement performed at least ≥48h before nosocomial infection diagnosis, documented NI and VAP**

|  | Only patients with mHLA-DR ≥48h before NI | | | Documented NI | | | VAP | | |
| --- | --- | --- | --- | --- | --- | --- | --- | --- | --- |
| variables | HR | IC 95% | p | HR | IC 95% | p | HR | IC 95% | p |
| HLA-DR_1_ < 8000 | 0.74 | [0.47 ; 1.18] | 0.21 | 1.03 | [0.69 ; 1.54] | 0.88 | 1.18 | [0.62 ; 2.25] | 0.62 |
| HLA-DR_2_ < 8000 | 1.36 | [0.87 ; 2.12] | 0.18 | 1.86 | [1.27 ; 2.74] | 0.00 | 1.19 | [0.66 ; 2.16] | 0.56 |
| Decreasing slope | 2.16 | [1.39 ; 3.36] | 0.00 | 1.57 | [1.07 ; 2.29] | 0.02 | 1.66 | [0.96 ; 2.86] | 0.07 |

HR: Hazard Ratio; IC: Confidence interval; HLA-DR: Human Leukocyte Antigen – antigen D Related. NI: nosocomial infection,
